# Supplementary material for: Role of Defects in Atom Probe Analysis of Sol−Gel Silica
Source: ACS Omega. 2025 Jul 22;10(30):33741–54. doi: 10.1021/acsomega.5c04733 (PMC12332786; doi:10.1021/acsomega.5c04733)
Supplement: Supplementary file 1 [file ao5c04733_si_001.pdf]

Role of defects in atom probe analysis of sol-gel silica

Gustav Eriksson,[*,†] Matteo De Tullio,[‡] Francesco Carnovale,[¶,§,‖] Giovanni Novi Inverardi,[¶,§,‖] Tommaso Morresi,[*,‖,§] Jonathan Houard,[⊥,‡] Marc Ropitaux,[⊥] Ivan Blum,[‡] Emmanuel Cadel,[‡] Gianluca Lattanzi,[¶,§] Mattias Thuvander,[#] Martin Andersson,[†] Mats Hulander,[†] Simone Taioli,[*,‖,§] and Angela Vella[*,‡]

† *Chalmers University of Technology, Department of Chemistry and Chemical Engineering, Kemigården 4, 412 96, Gothenburg, Sweden*

‡ *Université Rouen Normandie, INSA Rouen Normandie, CNRS, GPM UMR 6634, F-76000 Rouen, France*

¶ *Department of Physics, University of Trento, Via Sommarive 14, 38123, Trento, Italy*

§ *Trento Institute for Fundamental Physics and Applications (TIFPA), National Institute for Nuclear Physics (INFN), Via Sommarive 14, 38123, Trento, Italy*

‖ *European Centre for Theoretical Studies in Nuclear Physics and Related Areas (ECT*), Fondazione Bruno Kessler (FBK), Strada delle Tabarelle 286, 38122, Trento, Italy*

⊥ *Université Rouen Normandie, GLYCOMEV UR4358, SFR Normandie Végétal FED 4277, Innovation Chimie Carnot, IRIB, F-76000 Rouen, France*

Chalmers University of Technology, Department of Physics, Kemigården 1, 412 96, Gothenburg, Sweden

E-mail: gustav.eriksson@chalmers.se; morresi@ectstar.eu; taioli@ectstar.eu; angela.vella@univ-rouen.fr

PORES ANALYSIS

The isotherm of the silica sample is shown in Figure S.1. From this it can be deduced that the material contains both micropores and mesopores. The hysteresis loop observed in the multilayer range of the nitrogen sorption measurement is typical of silica gels and porous glasses. This hysteresis is due to a difference in the relative pressure required for adsorption or desorption. This is generally caused by a capillary condensation effect that depends on the pore radius of the material [S1]. An interpretation of this shape is to attribute it to the network effects of pores with narrow necks and wider bodies (in the form of ink bottles), where there is blockage of the pores that remain filled until the necks are emptied during desorption [S2]. It was found that the micropores have an average width of 6.8 Å and the mesopores have an average size of 40 Å.

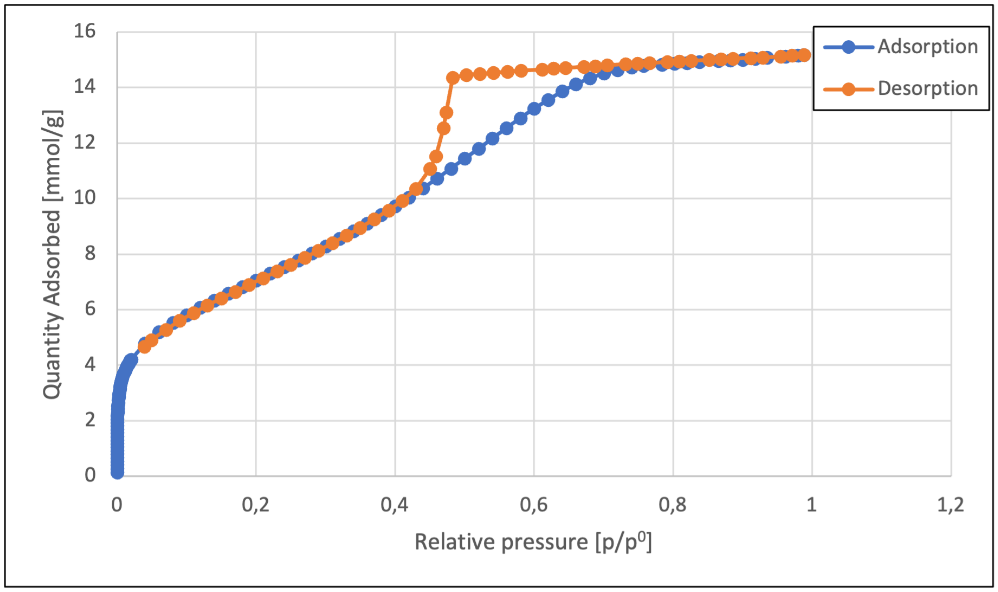

FIG. S.1. Complete isotherm measured on a representative silica sample.

UV/VIS SPECTROSCOPY OF SILICA

The absorbance of a thin silica sample in the UV/Vis range was measured with a Hewlett-Packard 8453 UV/Vis spectrometer, which was blanked against air. The absorption spectrum obtained is shown in Figure S.2. It can be seen from the spectrum that the relative absorbance increases gradually below 400 nm and more rapidly below 300 nm. The constant

measured absorbance above 400 nm is in part due to the scattering of light by the sample in addition to the absorption. Note that the sample is not perfectly flat, which contributes to the scattering of light.

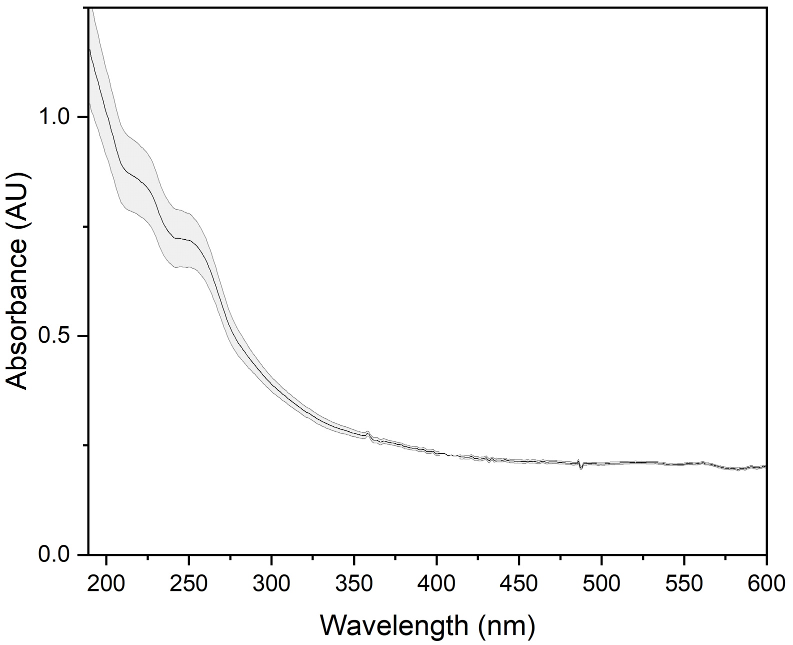

FIG. S.2. Absorption spectrum of a 0.6 mm thick silica sample that has been blanked against air. The standard deviation is shown in grey.

COMPUTATIONAL DETAILS OF ABSORPTION SPECTRA

In Figure S.3a we show a model of an amorphous silica matrix that we created using the MC method described in section 2.3 of the main text of the manuscript. In Figure S.3b we show its pair distribution with the typical peak centred at 1.6 Å representing the distance between Si and O atoms in amorphous silica matrices.

In Figure S.4 we show instead the imaginary part of the dielectric tensor for three different models of silicon dioxide corresponding to three different sizes of the boxes (yellow colour: $L = 2.14$ nm, N=648 atoms; green colour: $L = 1.42$ nm, N=192 atoms; red colour: $L = 1.03$ nm, N=72 atoms). We note that the spectrum in red is characterised by a spurious peak between 7 and 8 eV, which is not present when larger boxes are used. Conversely, while the

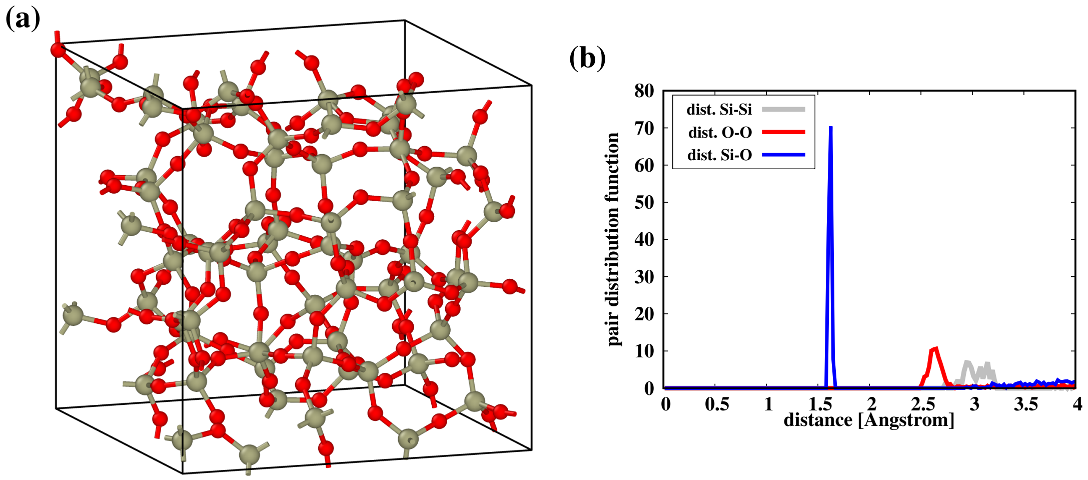

FIG. S.3. Model of the amorphous silica matrix: (a) SiO_2 cell with $N = 192$ atoms, corresponding to an edge of the box $L = 1.42$ nm. There are no defects in this structure. The red atoms are oxygen atoms, while the grey atoms are silicon atoms. (b) Corresponding pair distribution function.

details of the main peak around 10 eV are slightly different due to the different structures, the band gap of the two systems having $L = 1.42$ nm and $L = 2.14$ nm is the same. This shows us that a box size of $L = 1.42$ nm is sufficient to avoid spurious interactions between the periodic images when analysing the optical properties.

In Figure S.5 we report instead on the investigation of two different DFT functionals in the evaluation of the imaginary part of the dielectric tensor. A hybrid functional such as HSE06 reproduces the bandgap of amorphous silica much more accurately than the pure PBE exchange-correlation functional.

FITTING OF THERMAL TAILS IN TIME-OF-FLIGHT SPECTRA

To quantitatively investigate the thermal effects observed in the La-APT analyses in Fig. 4 of the article, we fitted the thermal tails of the Si^{2+} peak in the time-of-flight (ToF) spectra using equation (2) from the main text.

The fits were performed locally within a ∼100 ns window after the Si^{2+} peak maximum, which was selected to avoid interference from overlapping tails of subsequent peaks. Both ToF spectra were normalised to unity at the Si^{2+} peak and temporally aligned such that the onset of the thermal decay (t_0) overlapped. The initial estimates and boundaries for

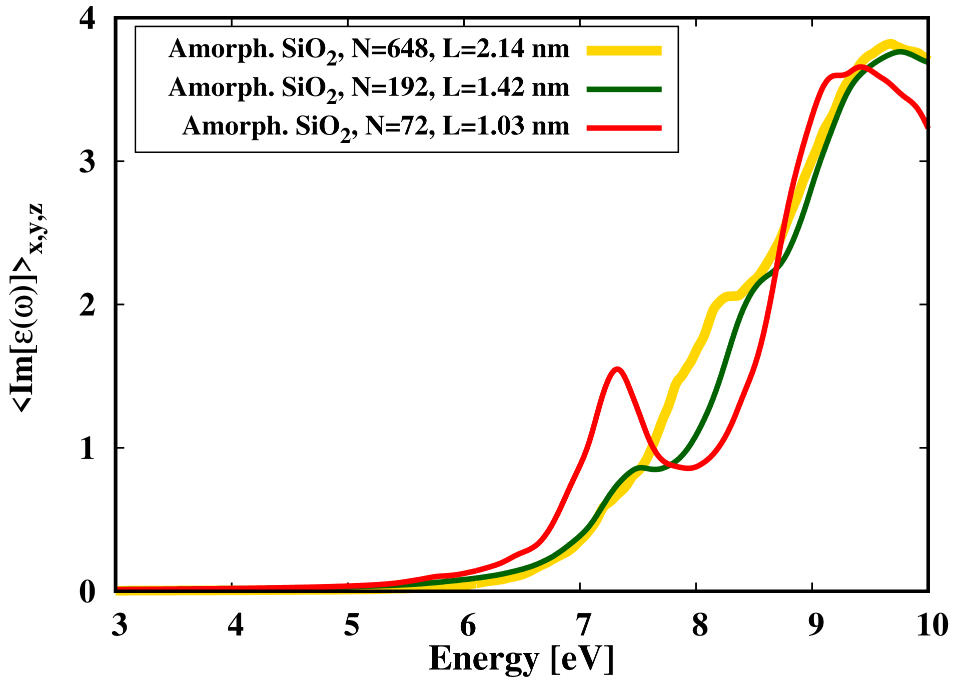

FIG. S.4. Effect of the finite size of the calculation supercell on the imaginary part of the dielectric tensor, averaged in the orthogonal Cartesian directions. Amorphous silica matrix without defects represented by a cell with $N = 648$ atoms corresponding to an edge of the box $L = 2.14$ nm (yellow colour) compared to a cell with $N = 192$ atoms corresponding to an edge of the box $L = 1.42$ nm (dark green colour) and to a cell with $N = 72$ atoms corresponding to an edge box of $L = 1.03$ nm (red colour). These calculations are performed with a PBE exchange-correlation functional.

the fitting parameters were physically motivated: Q was varied around 0.1 eV based on the expectations of CSR-derived electric fields; ΔT_{max} ranged from 65 K to 250 K and τ was constrained between 0.5 and 10 ns, depending on the typical values observed in the literature [S3, S4].

The fitting was performed by iterative parameter adjustment using OriginLab, aiming for a qualitative visual agreement between data and model. No automated statistical optimisation was performed; the fits are therefore considered illustrative. For the fit in Figure 4b of the main text, the extracted values of $Q_{\text{deep-UV}} = 0.12$ eV and $Q_{\text{UV}} = 0.06$ eV reflect the expectation that a higher electric field of 21.5 V/nm was used for UV analyses vs. 19.7 V/nm for deep-UV analyses. The values of 65K and 250 K for T_{max} were determined for the UV and deep-UV analyses, respectively.

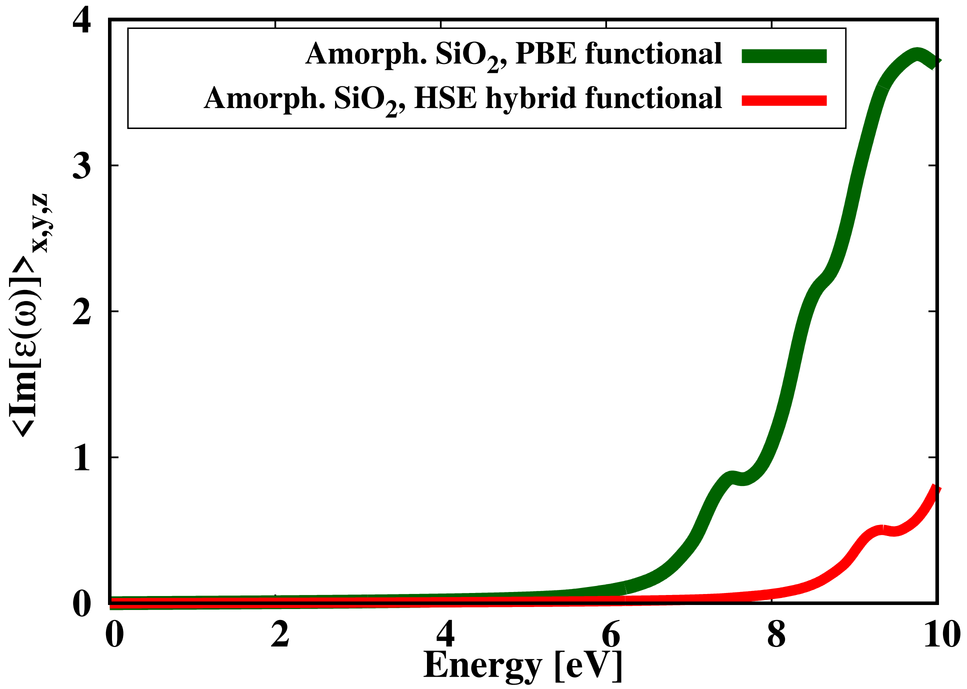

FIG. S.5. Effect of the DFT functional: average of the imaginary part of the dielectric tensor along the orthogonal Cartesian directions. Amorphous silica matrix using a PBE exchange-correlation functional (dark green colour) compared to a hybrid HSE06 functional (red colour).

EVOLUTION OF THERMAL BEHAVIOR DURING LEAP ANALYSIS

During the analysis with deep-UV light, the shape of the specimens changed, which is reflected in the change in their thermal response. Therefore, the ∼16 million ion dataset was split into two subsets: the first and the last 2 million events. The respective ToF spectra of the Si^{2+} peak were normalised and aligned as described above and then fitted separately. The data are shown in Fig. S.6. For the early phase of the analysis we obtained

- $Q = 0.14$ eV, $\Delta T_{\mathrm{max}} = 250$ K, $\tau = 6$ ns

For the late phase:

- $Q = 0.13$ eV, $\Delta T_{\mathrm{max}} = 250$ K, $\tau = 1.5$ ns

The significant reduction in τ indicates an increase in the cone angle during the course of the analysis, which is confirmed by the SEM image. T_{max} was kept constant for both fits because the illumination conditions are constant during the analysis and the value of Q is also almost constant.

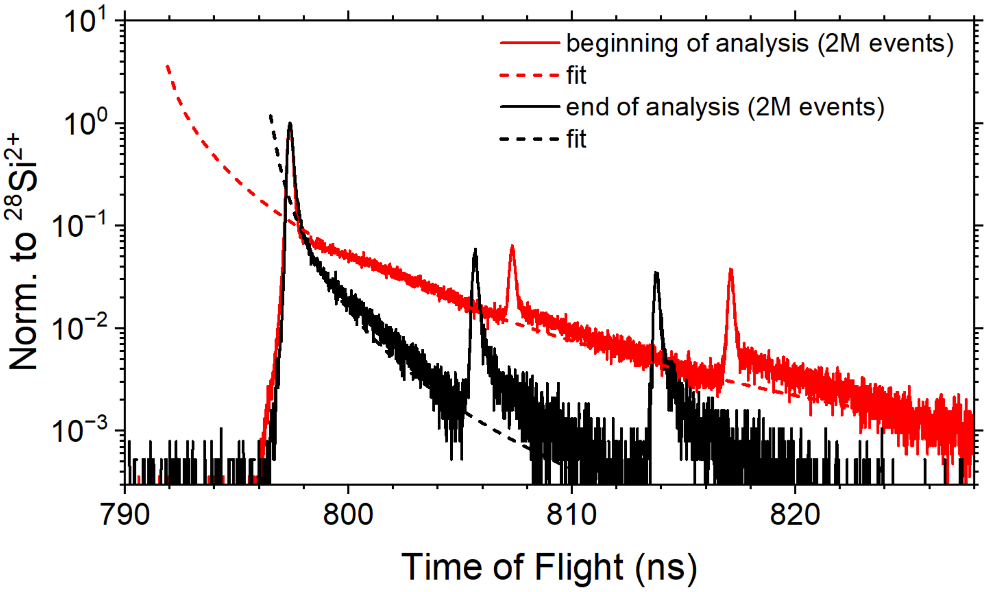

FIG. S.6. Time-of-flight (ToF) spectra of the Si^{2+} peak from the beginning (red line) and end (black line) of the LEAP analysis. The first 2 million events (black curve) and the last 2 million events (red curve) were extracted from a complete data set of ∼16 million ions. Both spectra were normalised to unity at the peak maximum and aligned so that the onset of thermal decay (t_0) overlaps. The curves were fitted using equation (2) from the main text. The optimal fitting parameters for the early phase were $Q = 0.14$ eV, $\Delta T_{\max} = 250$ K and $\tau = 6$ ns; for the late phase were $Q = 0.13$ eV, $\Delta T_{\max} = 250$ K and $\tau = 1.5$ ns.

DIELECTRIC PROPERTIES

In Figure S.7 we report the real and imaginary parts of the dielectric function for the three silicon dioxide samples of Figure 7a of the main text.

In Figure S.8 we show the refractive index of the silica sample 1 of Figure 7a of the main text.

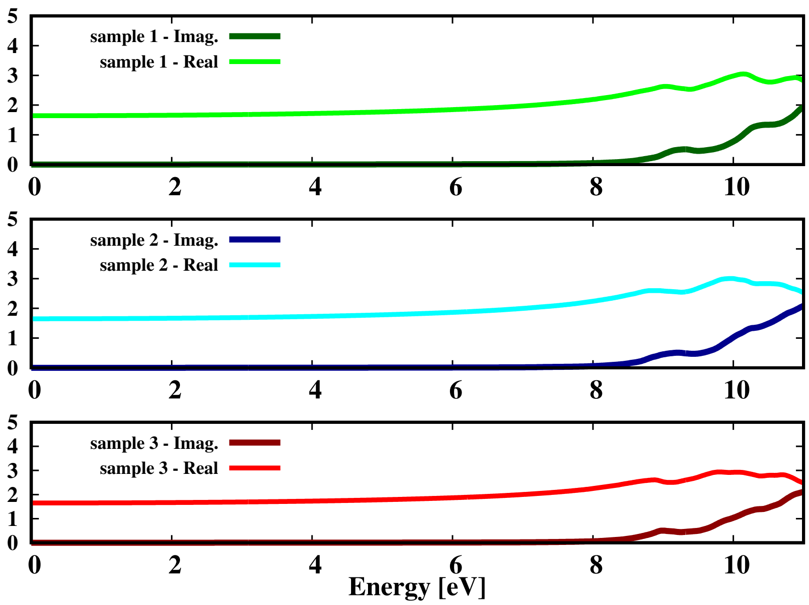

FIG. S.7. Real and imaginary part of the dielectric function for the three silicon dioxide samples in Figure 7a of the main text, calculated with the exchange-correlation functional HSE06.

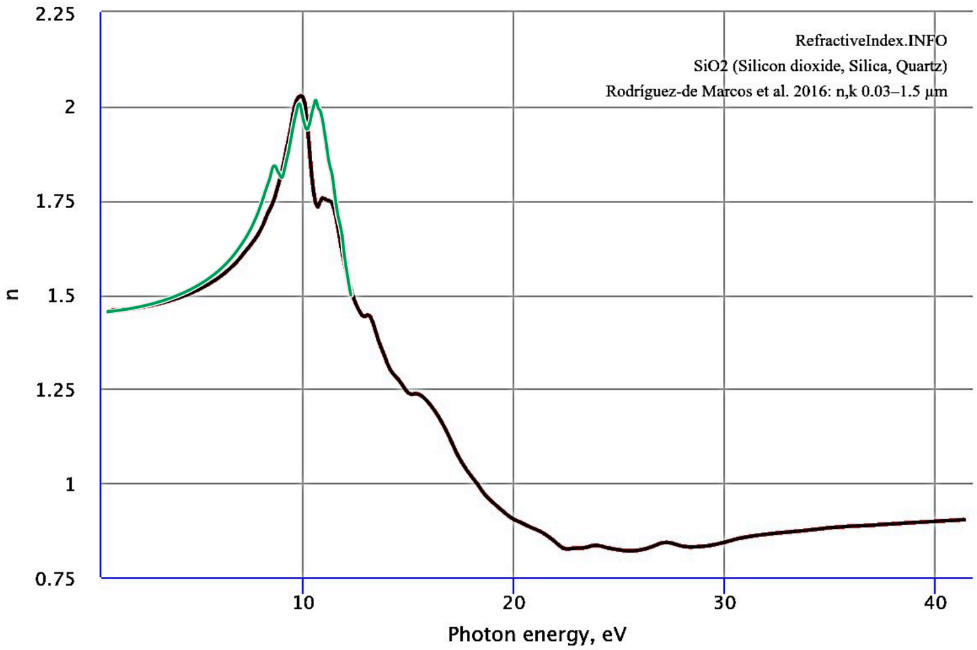

FIG. S.8. Refractive index of the silica sample 1 (green line), shown in Figure 7a of the main text, calculated with the exchange-correlation functional HSE06 in comparison to experimental data (black line).

[S1] K. S. W. Sing, Reporting physisorption data for gas/solid systems with special reference to the determination of surface area and porosity (recommendations 1984), Pure and Applied Chemistry **57**, 603 (1985).

[S2] M. Thommes, K. Kaneko, A. V. Neimark, J. P. Olivier, F. Rodriguez-Reinoso, J. Rouquerol, and K. S. Sing, Physisorption of gases, with special reference to the evaluation of surface area and pore size distribution (iupac technical report), Pure and Applied Chemistry **87**, 1051 (2015).

[S3] A. Vella, B. Mazumder, G. Da Costa, and B. Deconihout, Field evaporation mechanism of bulk oxides under ultra fast laser illumination, Journal of Applied Physics **110** (2011).

[S4] A. Vella, On the interaction of an ultra-fast laser with a nanometric tip by laser assisted atom probe tomography: A review, Ultramicroscopy **132**, 5 (2013).
